# Breath-hold spiral tissue phase velocity mapping (TPVM) with non-Cartesian SENSE

**DOI:** 10.1186/1532-429X-16-S1-P40

**Published:** 2014-01-16

**Authors:** Robin Simpson, Jennifer Keegan, Peter Gatehouse, Michael Hansen, David Firmin

**Affiliations:** 1 NIHR CBRU, Imperial College, London, UK; 2 CMR Department, Royal Brompton Hospital, London, UK; 3 National Heart, Lung and Blood Institute, NIH, Bethesda, Maryland, USA

## Background

TPVM is the only MR technique capable of measuring regional myocardial mechanics over the entire cardiac cycle [1,2]. Previous Cartesian breath-hold TPVM sequences have had low temporal resolution (eg [3]), limiting temporal detail, while navigator sequences are long and difficult to use clinically. This abstract presents a new spiral TPVM sequence which is capable of acquiring high temporal resolution TPVM data over the entire cardiac cycle within a clinically acceptable breath-hold duration.

## Methods

K-space is fully sampled with 8 spiral interleaves (14 ms duration, TR 24 ms) but only 3 spirals are acquired and reconstructed using non-Cartesian SENSE [4] implemented on the Gadgetron GPU framework [5]. Velocity compensated and encoded (30 cm/s through plane, 20 cm/s in-plane) data are acquired in consecutive heartbeats, with an initial heartbeat used to collect coil sensitivity information (total breath-hold duration 13 heartbeats). Acquired spatial resolution is 1.7 × 1.7 mm. Retrospective cardiac gating is used to cover the entire cycle (50 reconstructed phases). Basal, mid and apical short-axis slices were acquired in 10 volunteers and from the mid-slice of 1 early stage DCM patient (LVEF 49%, EDV 188 mL, ESV 96 mL) and one late stage DCM patient (LVEF 21%, EDV 326 mL, ESV 258 mL) on a Siemens Skyra 3T scanner. Global peak and time to peak (TTP) velocities were extracted. In one healthy volunteer a stack of 9 short axis slices were also acquired, providing full LV coverage.

## Results

**Figure 1:**
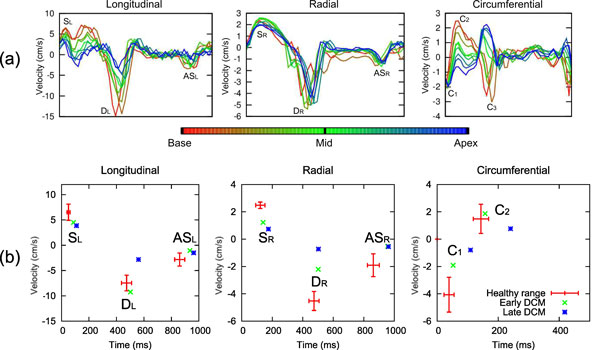
**a) Global velocities from 9 short axis slices in one volunteer (each curve represents velocity averaged over a slice)**. In longitudinal and radial directions one systolic (SL/SR), one early diastolic (DL/DR) and one atrial systolic (ASL/ASR) peak is seen. Longitudinal peaks reduce from base to apex, while radial peaks do not. Two systolic circumferential peaks (C1 and C2) are seen, as well as a diastolic peak (C3) which is negative at base but positive at apex. b) Peak and TTP global velocity values in the mid slice for the healthy volunteers (mean ± SD values shown by red bars), early DCM patient (green crosses) and late DCM patient (blue crosses). Healthy values show small standard deviations (although circumferential peaks are more variable). C3 is not shown as it is very slice-position dependent not always seen in the mid slice. The early stage patient shows normal longitudinal values but reduced radial values, whereas the late stage patient shows reduced peak values in all directions.

**Figure 2:**
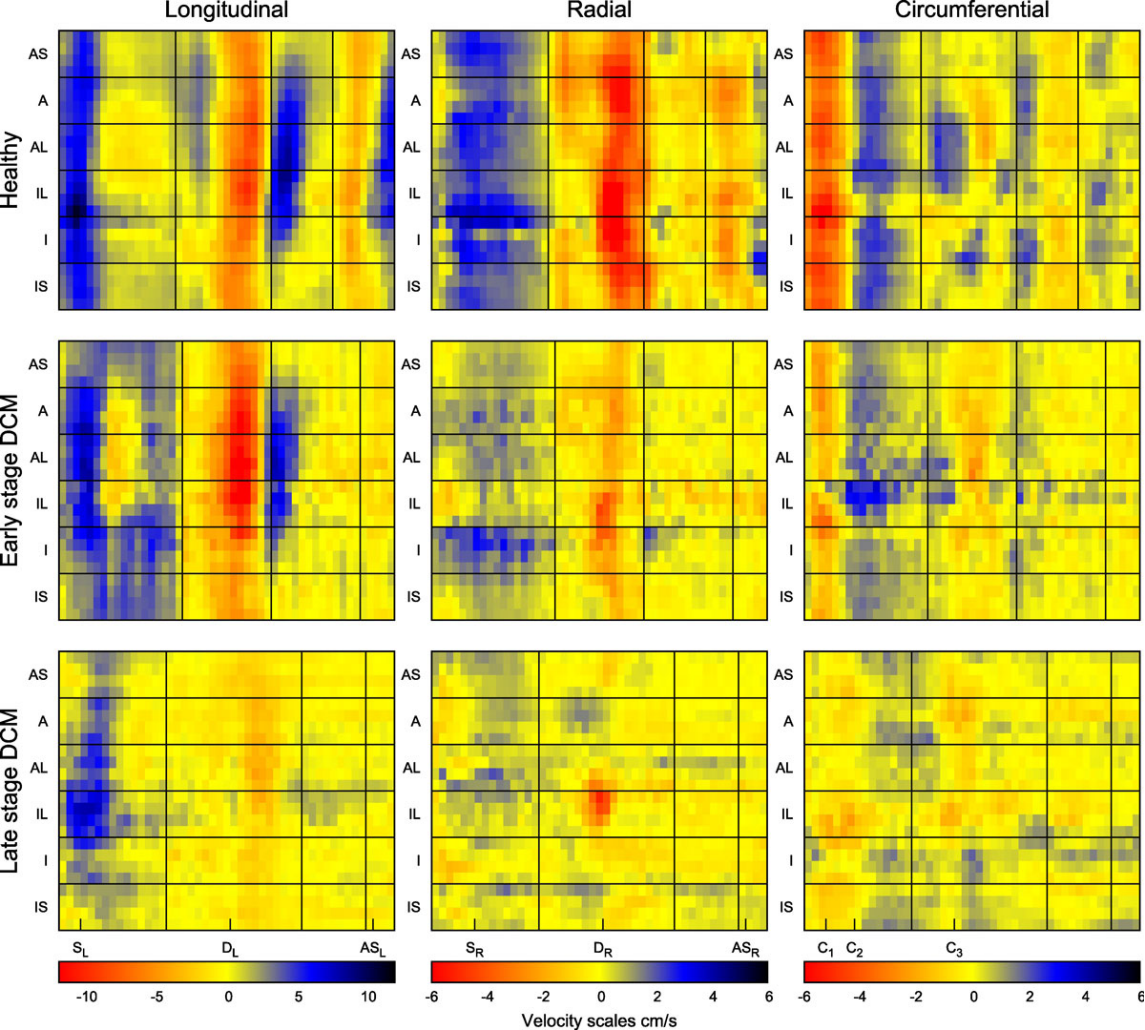
**Regional velocity colourplots for the mid slice of a healthy volunteer, the early DCM patient and the late DCM patient in the longitudinal, radial and circumferential directions, allowing analysis of regional motion**. The global velocity peaks labelled in Figure 1 are marked beneath. The longitudinal plot for the early DCM patient is similar to the healthy example but the radial direction shows lowered velocities throughout the cycle and abnormal regional variation (for example the early diastolic peak is heterogeneous across the myocardium). The late DCM example has normal longitudinal systolic velocities in the lateral region but lowered velocities elsewhere. The radial direction shows localised diastolic relaxation in the lateral regions and very low velocities elsewhere throughout the cardiac cycle. The circumferential direction is most slice dependent and most variable between the healthy volunteers (as shown in Figure 1) and so it is more difficult to assess.

Figure 1a shows global velocities measured from the 9 short axis slices in one volunteer with velocity peaks labelled. This shows smooth changes from base to apex that have only previously been seen in lengthy 3D acquisitions [5]. Figure 1b shows mid-slice healthy mean (+/- SD) peak and time to peak velocities as well as the individual patient values. Longitudinal peaks in the early DCM patient are normal but radial values are clearly reduced. For the late stage patient, velocities in all directions are reduced. Figure 2 shows regional velocity variation (y-axis) over time (x-axis) in the mid slice of one volunteer and both patients. Regional abnormalities can be seen in the radial direction of the early DCM example, although longitudinal velocities are normal. The late DCM patient has clearly abnormal regional variation in all directions, particularly in early diastole.

## Conclusions

Spiral trajectories and non-Cartesian SENSE has allowed acquisition of high temporal resolution TPVM images within a clinically achievable breath-hold and allows acquisition and reconstruction of a full short axis stack within 30 minutes. Initial clinical examples indicate that this technique is capable of detecting reduced radial velocities in early DCM when global parameters of motion are still within the normal range.

## Funding

HRUK Grant RG2584. NHLI CBRU Royal Brompton Hospital and Imperial College.
